# Supplementary material for: Mating activates neuroendocrine pathways signaling hunger in Drosophila females
Source: eLife. 2023 May 15;12:e85117. doi: 10.7554/eLife.85117 (PMC10229122; doi:10.7554/eLife.85117)
Supplement: Supplementary file 1. — Table including the short and full genotypes of flies in the indicated figure. [file elife-85117-supp1.docx]

**Table S1. Fly genotypes in figures**

**Figure   short genotype                  full genotype**

2b-d Gr64f-GAL4 > GCaMP         *UAS-CD8::tdTomato/+;20xUAS-IVS-GCaMP6s(attP40)/Gr64f-*

*GAL4; 20xUAS-IVS-GCaMP6s(VK00005)/Gr64f-GAL4*

3a    hs-bam                             *Hsp70(generic)-bam.O;+;+*

3c    fru-LexA ∩ ppk-GAL4 > GFP    *w*; 8xLexAop2-FLPL(attP40)/UAS(FRT.stop)Hsap\*

*mcd8::GFP(VIE-19a); Fru-PI-LexA/ppk-GAL4*

3d    fru-LexA ∩ ppk-GAL4 > TNT    *w+; 8xLexAop2-FLPL(attP40)/w[+m*]=UAS(FRT.stop)Ctet\*

*tetX(VIE-19A); Fru-PI-LexA/ppk-GAL4*

3d    fru-LexA, UAS-TNT (control)  *w+; w[+m*]=UAS(FRT.stop)Ctet\tetX(VIE-19A)/+; Fru-PI-LexA/+*

3e    fru-LexA ∩ ppk-GAL4 > KIR2.1 *w+;8xLexAop2-FLPL(attP40)/UAS(FRT.stop)Hsap\KCNJ2*

*(VIE-19A); Fru-PI-LexA/ppk-GAL4*

3e    fru-LexA, UAS-KIR (control)   *w+; UAS(FRT.stop)Hsap\KCNJ2(VIE-19A)/+; Fru-PI-LexA/+*

3d,e  ppk-GAL4 (control)                 *w+;8xLexAop2-FLPL(attP40)/+;ppk-GAL4/+*

3f     SAG-SS3 > GFP                   *w*;20xUAS-csChrimson::mVenus(attP18)/+; VT050405-*

*p65.AD(attP40)/+;dsx^DBD^/+*

3g    SAG-SS3 > GtACR1             *w+; VT050405-p65.AD(attP40)/+;20xUAS-IVS-GtACR1*

*EYFP(attP2)/dsx^DBD^*

3g    SAG-SS3 (control)                *w+; VT050405-p65.AD(attP40)/+; dsx^DBD^*/+

3h    pC1-SS1 > GFP                    *w*;20xUAS-csChrimson::mVenus(attP18)/+; VT002064-p65AD*

*(attP40)/+; VT008469-GAL4DBD(attP2)/+*

3i     pC1-SS1 > GtACR1              *w+; VT002064-p65AD(attP40)/+;20xUAS-IVS-GtACR1-EYFP*

*(attP2)/VT008469-GAL4DBD(attP2)*

3i     pC1-SS1 (control)                 *w+; VT002064-p65AD(attP40)/+; VT008469-GAL4DBD(attP2)/+*

3g,i   GtACR1 (control)                  *w+; +; 20xUAS-IVS-GtACR1-EYFP(attP2)/+*

4b    vpoDN-SS1 > GtACR1          *w+; 31D07-p65AD(attP40)/+;20xUAS-IVS-GtACR1*

*EYFP(attP252F12-GAL4DBD(attP2)*

4b    vpoDN-SS1 (control)             *w+; 31D07-p65AD(attP40)/+;52F12-GAL4DBD(attP2)/+*

4c    vpoDN-SS2 > GtACR1          *w+; VT045670-p65AD(attP40)/+; 20xUAS-IVS-GtACR1-EYFP*

*(attP2)/52F12-GAL4DBD(attP2)*

4c    vpoDN-SS2 (control)             *w+; VT045670-p65AD(attP40)/+;52F12-GAL4DBD(attP2)/+*

4b,c  GtACR1 (control)                  *w+; +; 20xUAS-IVS-GtACR1-EYFP(attP2)/+*

4d-f  oviDN-SS1 > csChrimson      *w*,20xUAS-csChrimson::mVenus(attP18)/+; VT050660-p65.AD*

*(attP40)/+; VT028160-GAL4DBD(attP2)/+*

4d-f  oviDN-SS1 (control)              *w*; VT050660-p65.AD(attP40)/+; VT028160-GAL4DBD(attP2)/+*

4d-g csChrimson (control)             *w*,20xUAS-csChrimson::mVenus(attP18)/+;+*

4d,e,g   oviDN-SS2 > csChrimson      *w*,20xUAS-csChrimson::mVenus(attP18)/+; VT026873-p65.AD*

*(attP40)/+; VT040574-GAL4DBD(attP2)/+*

4d,e,g   oviDN-SS2 (control)              *w*; VT026873-p65.AD(attP40)/+; VT040574-GAL4DBD(attP2)/+*

5e    pCd-2a-SS1 > GFP                *w*,20xUAS-csChrimson::mVenus(attP18)/+; VT027804-p65.AD*

*(attP40)/+; dsx^DBD^/+*

5f     pCd-2b-SS1 > GFP                *w*,20xUAS-csChrimson::mVenus(attP18)/+; R9B05-p65.AD*

*(attP40)/+; R22D06-GAL4DBD(attP2)/+*

5g    pC1-SS1 > Chrimson,           *w*,10xUAS-syn21-opGCaMP6s,LexAop2-syn21-Chrimson88-*

pCd-2a-SS1 > GCaMP6s                  *tdTomato; VT027804-p65AD(attP40)/R40F04-LexA(attp40);*

*dsx^DBD^/+*

5h    pC1-SS1 > Chrimson,           *w*,10x-UAS -syn21-opGCaMP6s, LexAop2-syn21-Chrimson88-*

pCd-2b-SS1 > GCaMP6s                 *tdTomato; R9B054-p65AD(attP40)/R40F04-LexA(attp40);*

*R22D06-GAL4DBD(attP2)/+*

5i,k pCd-2a-SS1 > GtACR1           *w+; VT027804-p65AD(attP40)/20xUAS-IVS-GtACR1-EYFP*

*(attP2)/dsx^DBD^*

5i,k  pCd-2a-SS1 (control)              *w+; VT027804-p65AD(attP40)/+; dsx^DBD^/+*

5j,k   pCd-2b-SS1 > GtACR1           *w+; R9B05-p65AD(attP40)/20xUAS-IVS-GtACR1-EYFP(attP2)/*

*R22D06-GAL4DBD(attP2)/+*

5j,k pCd-2b-SS1 (control)              *w+; R9B05-p65AD(attP40)/+; R22D06-GAL4DBD(attP2)/+*

5i-k GtACR1 (control)                  *w+; +; 20xUAS-IVS-GtACR1-EYFP(attP2)/+*

6c,g    Lgr3 ∩ Fru > CsChrimson       *w-,20xUAS(FRT.stop)csChrimson:mVenus(attP18)/+;*

*8xLexAop2-FLPL(attP40)/+; Fru-PI-LexA/R19B09-GAL4*

6d    pCd-2a-SS1 > Chrimson,        *w*,LexAop2-syn21-opGCaMP6s,10x-UAS-syn21-Chrimson88-*

Lgr3 > GCaMP6s                  *tdTomato; VT027804-p65AD(attP40)/R19B09-LexA(attp40);*

*dsx^DBD^/+*

6e     pCd-2b-SS1 > Chrimson,        *w*,LexAop2-syn21-opGCaMP6s,10x-UAS-syn21-Chrimson88-*

Lgr3 > GCaMP6s                  *tdTomato; R9B05-p65AD(attP40)/R19B09-LexA(attp40);*

*R22D06-GAL4DBD(attP2)/+*

6d,e  control                                  *w*,LexAop2-syn21-opGCaMP6s,10x-UAS-syn21-Chrimson88-*

*tdTomato; R19B09-LexA(attp40)/+;+*

6g CsChrimson, Fru-LexA (control) *w-,20xUAS(FRT.stop)csChrimson:mVenus(attP18)/+;*

*8xLexAop2-FLPL(attP40)/+; Fru-PI-LexA/+*

6g R19B09-GAL4 (control) w-;+; *R19B09-GAL4/+*
